# Genome-wide selection signal analysis reveals the adaptability of Tibetan sheep to high altitudes

**DOI:** 10.3389/fvets.2025.1632017

**Published:** 2025-08-14

**Authors:** Yufang Song, Chao Yuan, Tingting Guo, Bowen Chen, Fan Wang, Zengkui Lu, Jianbin Liu

**Affiliations:** ^1^Key Laboratory of Animal Genetics and Breeding on Tibetan Plateau, Ministry of Agriculture and Rural Affairs, Lanzhou Institute of Husbandry and Pharmaceutical Sciences, Chinese Academy of Agricultural Sciences, Lanzhou, China; ^2^Sheep Breeding Engineering Technology Research Center of Chinese Academy of Agricultural Sciences, Lanzhou, China; ^3^College of Life Science and Engineering, Northwest Minzu University, Lanzhou, China

**Keywords:** Tibetan sheep, high-altitude adaptation, whole-genome resequencing, Fst, θπ ratio, selection signal

## Abstract

Altitude adaptation is a complex process involving multiple physiological and biochemical responses to hypoxia and other environmental stresses. In-depth genetic analysis of Tibetan sheep, which exhibit significant adaptations to high-altitude hypoxia, promises to elucidate hypoxia-tolerance mechanisms in plateau animals. Here, we conducted a genome-wide selection scan on three Tibetan sheep populations: low-altitude Tao (TS; 2887 m), medium-altitude Tianjun white (WT; 3331 m), and high-altitude Huoerba (HB; 4614 m). Using the population differentiation index (Fst) and nucleotide diversity (θπ) ratio, we analyzed selection signals associated with hypoxia at high-altitudes. We screened 865, 941, and 876 candidate genes in the TS vs. WT, TS vs. HB, and WT vs. HB group comparisons, respectively, 55 of which were jointly screened. Integrated analysis further identified several key pathways and genes under positive selection in Tibetan sheep populations, including metabolic pathways (*GSTA1*, *ALAS1*, *HMOX2*, *SCD*, *ME1*, *ACSL6*, *PIK3C2G*), melanogenesis (*MITF*, *EP300*), and the HIF-1 signaling pathway (*ERBB2*, *HIF1A*, *RELA*). Among these, the metabolic pathways may enhance energy production under hypoxic conditions, while melanogenesis and the HIF-1 signaling pathway are likely associated with ultraviolet radiation protection and hypoxia tolerance, respectively. This study provides valuable insights into the genetic mechanism of high-altitude adaptation in Tibetan sheep, and also provides important theoretical basis for the conservation and breeding of Tibetan sheep and the sustainable development of plateau animal husbandry.

## Introduction

The Tibetan Plateau is the largest and highest plateau in the world, with an average elevation exceeding 4000 m. This extreme environment is characterized by low oxygen levels, low atmospheric pressure, low temperatures, and intense ultraviolet (UV) radiation (1). High-altitude regions are significant socioeconomic habitats, particularly for ruminant livestock, which serve as a primary source of food and income for local communities. However, because of harsh environmental conditions and limited forage resources, ruminants face severe survival challenges (2). Nevertheless, some ruminant species, such as goats (3, 4), sheep (5), and yaks (6, 7), demonstrate remarkable adaptive capabilities, enabling them to thrive in these extreme environments. One important ruminant of this landscape, the Tibetan sheep (*Ovis aries*), has evolved a hypoxia-adaptive mechanism that deserves special attention.

As one of the three major coarse-wool sheep breeds in China, Tibetan sheep primarily inhabit plateau regions characterized by extreme conditions, such as severe cold, hypoxia, and intense UV radiation. They survive in harsh environments with remarkable adaptability, providing essential resources (e.g., meat, wool, and leather) that play a vital role in the development of local animal husbandry practices (8). Through long-term natural selection and artificial breeding, Tibetan sheep have developed unique anatomical and physiological traits to thrive in this habitat, making them an ideal animal model for studying high-altitude hypoxia adaptation (9).

In recent years, rapid advances in high-throughput sequencing technology have spurred the widespread use of whole-genome resequencing. Specifically, detecting whole-genome selection signals has become a crucial research area, precisely revealing which genes and their genomic regions have been significantly influenced by natural selection or artificial breeding. In-depth analysis of selective signals facilitates a comprehensive understanding of the genetic traits that species develop during adaptation to natural environments. It also allows for the precise identification of genetic factors closely related to production traits, which can be used in animal genetic breeding programs to enhance production performance (10). Selection signal analysis has become an important research method for unraveling the genetic mechanisms underlying important economic traits in Tibetan livestock, such as sheep, goats, and horses. For instance, it has led to the identification of multiple genes associated with high-altitude adaptation, including *EPAS1* in Tibetan horses (11), *FGF10*, *MMP14*, *SLC25A51*, *ALAS1*, *PRMT1*, *HMOX2*, and *HIF1AN* in Tibetan sheep (5), and *FGF2*, *EGFR*, *AKT1*, *PTEN*, *MITF*, *ENPEP*, *SIRT6*, *KDR*, and *CDC42* in Tibetan goats (4). Additionally, *EPAS2*, which is associated with plateau adaptation, and *IRF2* and *EXOC2*, which are related to coat color, have been identified in goat breeds (12), further deepening our understanding of their adaptation mechanisms.

Livestock play a crucial role in human society. These domesticated animals were originally domesticated from wild animals. Through long-term natural selection and artificial breeding, they gradually evolved into diverse breeds that are adapted to local environments and meet human needs. During this process, distinct selection signatures have been accumulated in their genomes (13). By analyzing these selective signals, functional genes related to important traits can be identified, providing a theoretical basis for breed improvement. Tibetan sheep exhibit remarkable adaptability to extremely high-altitude environments, demonstrating excellent survival and reproduction performance. In this study, we investigated three populations of Tibetan sheep from different altitudes: Tao (TS, 2887 m), Tianjun White Tibetan (WT, 3331 m), and Huoerba (HB, 4614 m). Whole-genome resequencing was used to assess population differentiation via population differentiation index (Fst) values and nucleotide diversity (θπ) ratios, enabling selection signal analysis. The results of this study provide a scientific basis for sheep breeding and husbandry strategies that aim to improve breed adaptation to different altitudes, thereby enhancing production efficiency and economic benefits.

## Materials and methods

### Ethics statement

All experimental studies involving sheep were approved by the Animal Ethics Committee at the Lanzhou Institute of Husbandry and Pharmaceutical Sciences, Chinese Academy of Agricultural Sciences (no. 20231447).

### Sample collection and resequencing

This study comprised 60 individuals of three Tibetan sheep breeds: 20 TS, 20 WT, and 20 HB, which were randomly selected to ensure representative sampling and minimize selection bias (Table 1). Blood samples were collected from all 60 sheep via jugular venipuncture into EDTA anticoagulant tubes and stored at −20°C for future use. DNA was extracted using a blood genome extraction kit (Tiangen Biotech Co. Ltd., Beijing, China). The quality of the DNA was assessed using a Nanodrop 2000 spectrophotometer (Thermo, Waltham, MA, USA) and agarose gel electrophoresis. Whole-genome resequencing was performed using the Illumina HiSeq X10 PE150 platform, and the data were used for subsequent analyses. All sequencing was performed by Guangzhou Gidio Biotechnology Co., Ltd. (China).

**Table 1:** Sample collection information.

Breed	Abbr.	Age	Size	Location	Altitude
Tao sheep	TS	Adult	20	Liulin Town, Zhuoni County, Gannan Tibetan Autonomous Prefecture, Gansu Province	2887 m
Tianjun White Tibetan sheep	WT	Adult	20	Xinyuan Town, Tianjun County, Haixi Mongolian and Tibetan Autonomous Prefecture, Qinghai Province	3331 m
Huoerba sheep	HB	Adult	20	Rima Village, Zhongba County, Xigaze City, Tibet Autonomous Region	4614 m

### Whole-genome sequence alignment and genetic variation detection

The raw sequencing reads were evaluated and filtered to ensure the accuracy of bioinformatics analysis, with the resulting data stored in FASTQ format. The filtering steps were as follows: (1) removal of adapters, retaining the remaining reads; (2) removal of reads containing >10% ‘N’ bases; and (3) removal of low-quality reads (number of bases with a quality value Q ≤ 20 accounting for more than half of the entire read).

The reference genome chosen for the comparison was Self-assembled genome_HB. High-quality sequencing data were aligned to the reference genome using BWA software (v 0.7.15; mem algorithm parameters: -k 32 -M) (14), and converted to BAM format using SAMtools (v 1.17) (15). Duplicate reads were then marked using Picard tool (v 2.18.7).^1^ Sequencing depth and coverage were determined using BEDTools statistics (16). Whole-genome resequencing was performed on 60 Tibetan sheep, yielding an average sequencing depth of 6.2 × (Supplementary Table S1); at depths of 1 × and 4×, the proportions of effective data were 92.50 and 63.83%, respectively (Supplementary Table S2).

To enhance the accuracy of data analysis, we filtered the dataset of single-nucleotide polymorphism (SNP) sites to exclude those that might compromise the accuracy of subsequent analyses. The quality control criteria were as follows: (1) SNPs with a call rate > 95% were selected; (2) Hardy–Weinberg equilibrium was verified, and SNPs with *p* > 0.0001 were retained; (3) SNPs with a minor allele frequency (MAF) > 0.05 were selected; (4) SNPs with unclear chromosomal localization were excluded, and sites on autosomal chromosomes were selected for subsequent analysis.

### Population structure analysis

Using PLINK software (v 1.09) (17), we pruned all single SNPs to obtain an independent set of SNP markers and conducted principal component analysis (PCA) to examine the clustering patterns within populations. To assess the genetic relatedness among individuals, we constructed a neighbor-joining (N-J) tree using Treebest software (18) and visualized it using iTOL (v6) software (19).^2^ To gain further insights into the evolutionary process, we estimated the ancestral population structure for K values ranging from 2 to 4 using ADMIXTURE software (v 1.3) (20), and visualized the results using Excel 2010 software.

### Selection signal analyses

Selection signal analysis plays a crucial role in the study of the evolution of biological populations by revealing the genomic traces of changes in phenotypic traits due to natural or artificial selection. Such traces generally manifest as increased genotypic homozygosity and reduced polymorphism at certain gene loci and fragments. To assess the degree of genetic differentiation between populations, we employed VCFtools (v 0.1.15) (21) to calculate Fst values and θπ ratios, identifying regions exhibiting strong selection signals. The range of Fst values was 0 to 1, with 0 indicating complete genotypic identity between two populations (i.e., no differentiation) and 1 indicating complete differentiation between them. The θπ ratio is primarily used to analyze nucleotide polymorphism, due to selective elimination in populations undergoing selection, polymorphism at certain loci decreases and homozygosity increases, so a smaller θπ value indicates lower nucleotide polymorphism and a higher degree of selection. Based on the screened SNPs, PopGenome software (22) was used to perform intrapopulation Fst and θπ ratio analyses, employing a sliding window with a physical length of 100 kb and a step size of 10 kb (23).

### Detection and annotation of candidate genes

Candidate selection loci were identified by screening windows in the top 5% based on Fst values and θπ ratios, filtering overlapping SNPs within these windows. The 50 kb regions upstream and downstream of these candidate loci were defined as selection signal regions (23). ANNOVAR software (24) was used for genomic annotation of candidate loci, and a Venn diagram based on the candidate genes was constructed.

### Candidate gene enrichment analysis

Enrichment analysis aims to deeply explore the functional roles and biological pathways associated with candidate genes. Using the powerful tools of the DAVID 6.8 databases^3^ and Kobas 3.0,^4^ we performed exhaustive Gene Ontology (GO) functional enrichment analysis and Kyoto Encyclopedia of Genes and Genomes (KEGG) pathway enrichment analysis on the annotated gene candidates. These analyses were conducted using *Ovis_aries* as the background organism, with *p* < 0.05 set as the significance threshold. To gain a deeper understanding of the results, we also searched the NCBI database^5^ for specific functional descriptions of related GO terms and KEGG pathways, screening out functional genes closely related to altitude traits in Tibetan sheep.

## Results

### Genetic variation

**Figure 1:**
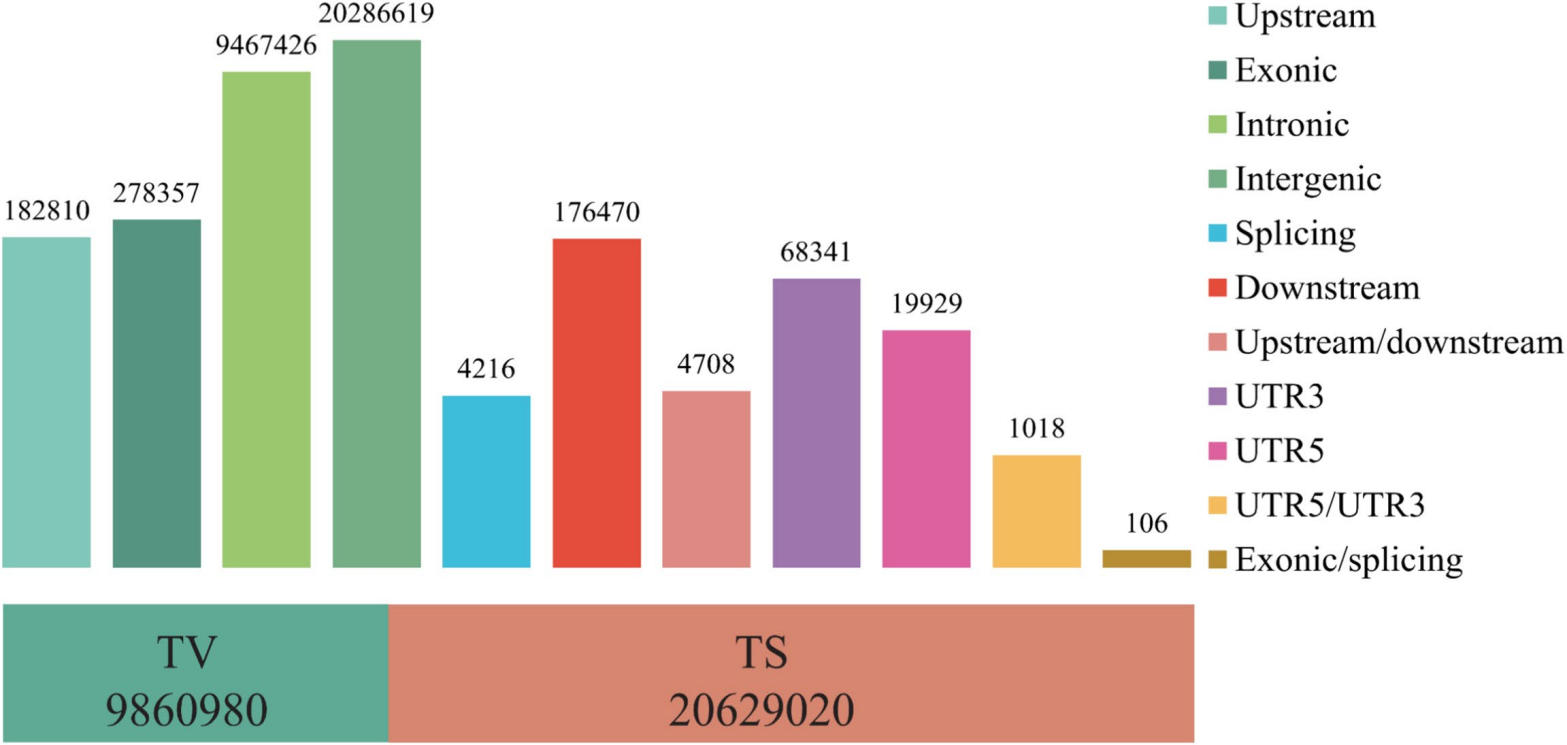
Distribution of SNP variations within genomic regions.

Firstly, the quality control was performed on the whole genome resequencing data of the 60 Tibetan sheep, and a total of 112,247,423 high-quality reads (16,237,358,665 bp) were obtained after quality control. Comparison with the reference genome_HB resulted in the identification of 30,490,000 SNPs. Genomic annotation revealed that these SNPs are mainly located in intergenic regions (20,286,619), followed by introns (9,467,426) and exon regions (278,357) (Figure 1). The transition/transversion (TS/TV) ratio was 2.02, indicating standardization of the genomic population structure. After filtering SNPs, there were 2,374,551, 2,466,610, and 2,425,773 SNPs in the TS, WT, and HB groups, respectively. The number of SNPs shared by the TS vs. WT, TS vs. HB and WT vs. HB group pairings was 2,296,612, 2,262,003, and 2,344,376, respectively. These findings provided a reliable foundation for further examination of the population structure and the identification of potential selection signals.

### Population genetic analysis

**Figure 2:**
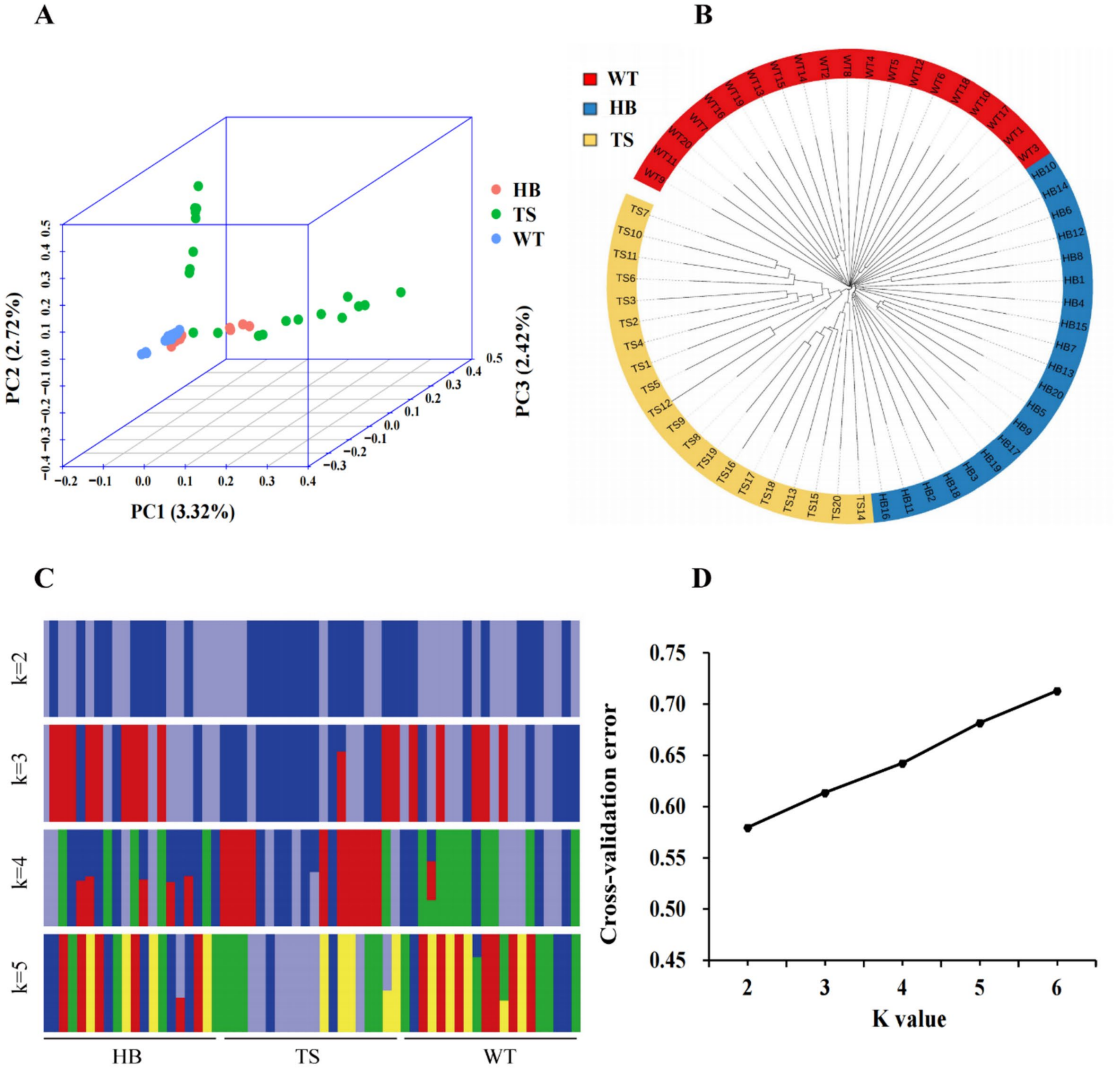
Analysis of the population genetic structure. **(A)** Principal component analysis (PCA). **(B)** Phylogenetic tree generated using the neighbor-joining method. **(C)** Population structure analysis at K = 2, 3, 4, or 5. Different colors represent different components of ancestry. **(D)** Cross-validation error.

Figure 2A shows the results of PCA on three Tibetan sheep populations. PC1 accounted for 3.32% of the genetic variation, while PC2 and PC3 explained 2.72 and 2.42%, respectively. PC1 and PC2 separated the TS population, while PC3 showed a certain degree of overlap among the three populations. The PCA results revealed the distribution in genetic variation to be relatively compact, with the WT and HB populations clustered closely together, and the TS population more distantly separated. In the N-J tree (Figure 2B), the three populations were grouped into three branches, with the WT and HB groups forming a close cluster, mirroring the results in the PCA. The cross-validation error suggested that a K value of 2 may be optimal for modeling (Figure 2D). Population genetic structure analysis showed that, when *K* = 2 (Figure 2C), the TS population displayed a distinct, darker blue component, separated from the other two, which aligned well with the results of PCA and the N-J tree. This reinforced the reliability of the approach used to analyze the population genetic structure.

### Analysis of selection signals

**Figure 3:**
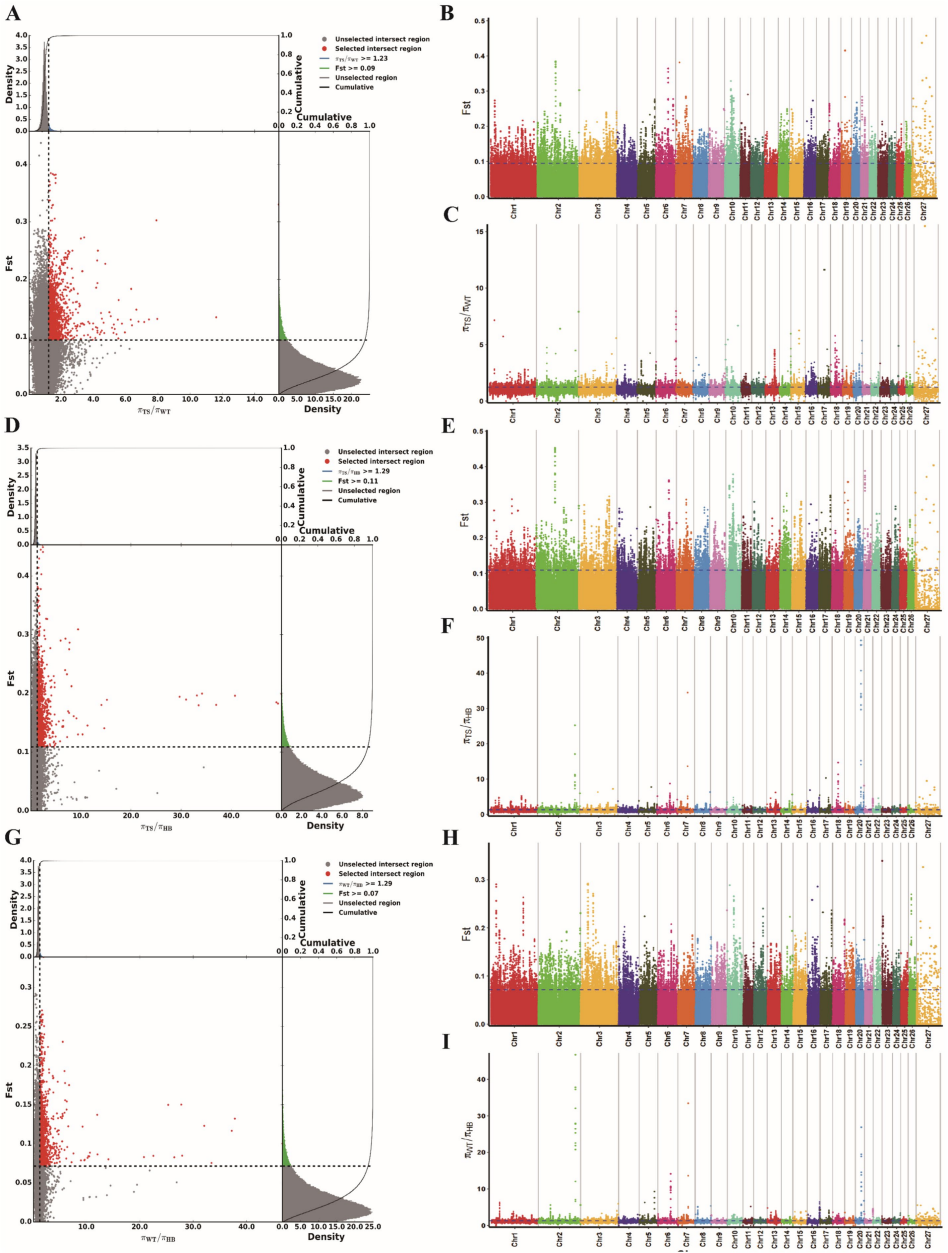
Analysis of selection signals. **(A)** Fst and θπ ratio joint selection elimination (TS vs. WT). **(B,C)** The genome-wide distribution of Fst and θπ Ratio (TS vs. WT). **(D)** Fst and θπ ratio joint selection elimination (TS vs. HB). **(E,F)** The genome-wide distribution of Fst and θπ Ratio (TS vs. HB). **(G)** Fst and θπ ratio joint selection elimination (WT vs. HB). **(H,I)** The genome-wide distribution of Fst and θπ Ratio (WT vs. HB).

Using two different selection analysis methods, Fst and θπ ratio, we screened for strongly selected genetic loci, identifying 2438, 2810, and 3057 loci in the TS vs. WT, TS vs. HB, and WT vs. HB group comparisons, respectively (Figures 3A–I). These SNP loci were annotated to 865, 941 and 876 candidate genes, respectively (Supplementary Figure S1; Supplementary Table S3). Among these, we identified 313 common candidate genes between the TS vs. WT and TS vs. HB comparisons, 96 between TS vs. WT and WT vs. HB, and 314 between TS vs. HB and WT vs. HB. Additionally, 55 candidate genes overlapped across all three groups.

### Enrichment analysis of candidate genes

**Figure 4:**
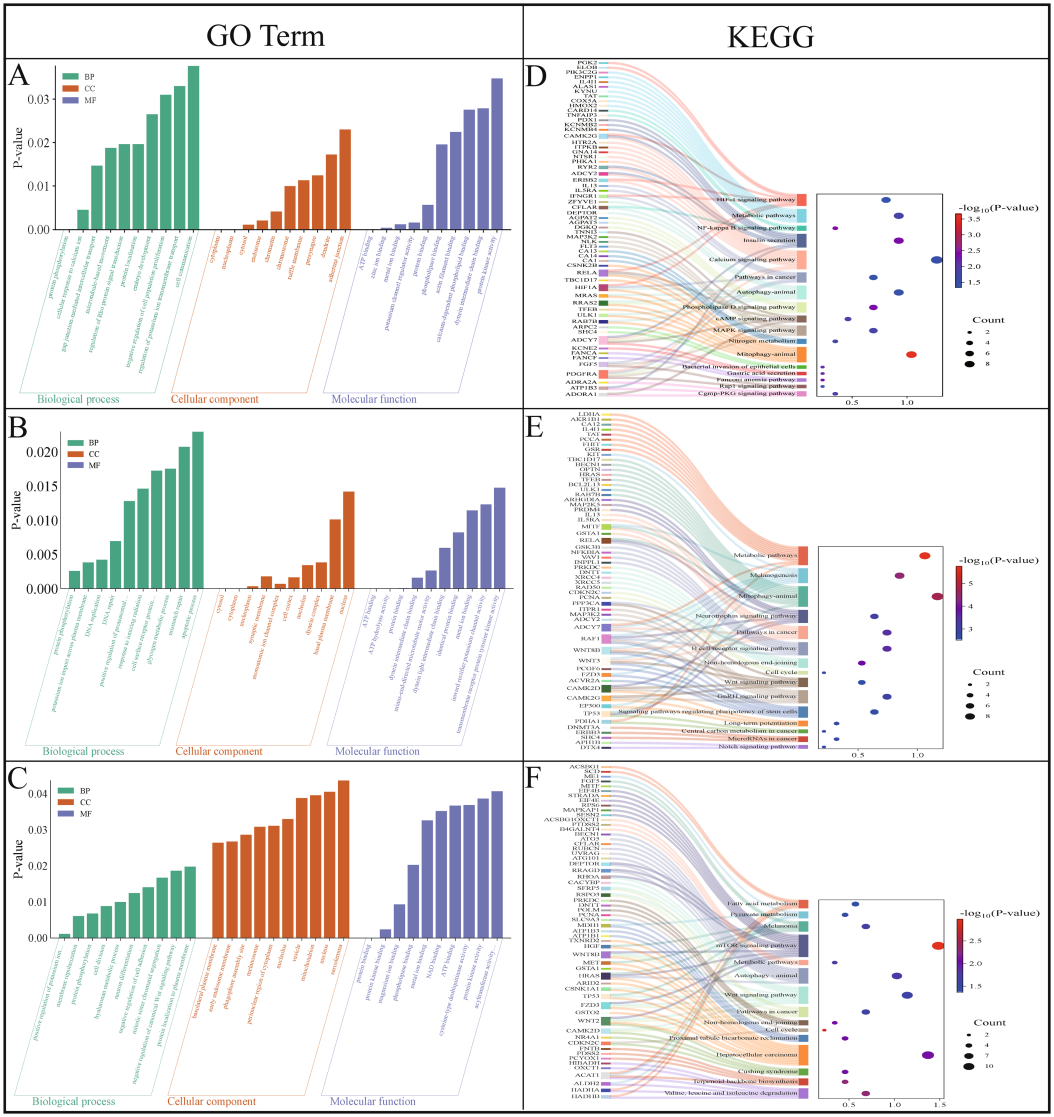
GO enrichment and the KEGG pathway enrichment analyses. **(A)** GO terms enriched in TS vs. WT. **(B)** GO terms enriched in TS vs. HB. **(C)** GO terms enriched in WT vs. HB. **(D)** TS vs. WT enriched the KEGG pathway. **(E)** TS vs. HB enriched the KEGG pathway. **(F)** WT vs. HB enriched the KEGG pathway.

GO enrichment analysis revealed significant enrichment (*p* < 0.05) of 43 GO terms in the TS vs. WT comparison, including altitude adaptation-related categories, such as cellular response to calcium ion (GO:0071277), protein localization (GO:0008104), and protein binding (GO:0005515) (Figure 4A; Supplementary Table S4). In the TS vs. HB (Figure 4B; Supplementary Table S5) and WT vs. HB (Figure 4C; Supplementary Table S6) comparisons, there were 62 and 49 significantly enriched GO terms, respectively. Those associated with altitude adaptation included protein phosphorylation (GO:0006468), glycogen metabolic process (GO:0005977), negative regulation of canonical Wnt signaling pathway (GO:0090090), ATP binding (GO:0005524), melanosome (GO:0042470), magnesium ion binding (GO:0000287), and actin cytoskeleton (GO:0015629).

In the KEGG enrichment analysis of candidate genes in the TS vs. WT comparison, 40 pathways were significantly enriched (*p* < 0.05), including the following altitude-associated pathways: HIF-1 signaling (oas04066), metabolic (oas01100), NF-kappa B signaling (oas04064), and calcium signaling (oas04020) (Figure 4D; Supplementary Table S7). In the TS vs. HB comparison, 22 pathways were significantly enriched, including high-altitude adaptation-related pathways involving GnRH signaling (oas04912), B-cell receptor signaling (oas04662), Wnt signaling (oas04310), and melanogenesis (oas04916) (Figure 4E; Supplementary Table S8). Among the 31 significantly enriched pathways in the WT vs. HB comparison, those associated with high-altitude adaptation included mTOR signaling (oas04150), melanoma (oas05218), fatty acid metabolism (oas01212), Wnt signaling (oas04310), cancer (oas05200), and metabolic (oas01100) (Figure 4F; Supplementary Table S9).

Among all the significantly enriched pathways, a relatively large number of genes were involved in metabolic pathways. We identified candidate genes including *GSTA1*, *ALAS1*, *HMOX2*, *SCD*, *ME1*, *ACSL6*, and *PIK3C2G* that are closely associated with energy metabolism in Tibetan sheep. These genes were primarily enriched in the Metabolic pathways category, suggesting that Tibetan sheep may adapt to the hypoxic and low-temperature conditions of the plateau environment by regulating energy metabolism. Additionally, we found that genes such as *ERBB2*, *HIF1A*, and *RELA*, which are enriched in the HIF-1 signaling pathway, are related to hypoxia tolerance. This indicates that Tibetan sheep may enhance their hypoxia tolerance by activating the HIF-1 signaling pathway. Furthermore, *MITF* and *EP300* genes were mainly enriched in the Melanogenesis pathway, suggesting that Tibetan sheep may protect themselves against UV damage by enhancing melanin production, thereby adapting to the high-altitude environment.

## Discussion

Extreme environments marked by hypoxia, low temperatures, and UV radiation pose significant survival challenges for plateau-dwelling organisms, limiting the development of animal husbandry in these regions. In response to these environmental pressures, animals on the Tibetan Plateau have evolved unique morphological and physiological characteristics. Tibetan sheep are an important source of germplasm on the Tibetan Plateau, featuring strong adaptability to cold and hypoxic environments and serving as an important economic asset for farmers and herders in the region. Native animals, such as Tibetan sheep, serve as excellent models for studying the molecular regulatory mechanisms involved in adaptation to high-altitude environments. Therefore, to reveal genome-wide selection signals related to altitude adaptation, we conducted whole-genome resequencing of three Tibetan sheep populations from different altitudes. A sequencing depth of 6.2 × enhanced the reliability of variant annotation, mutation site identification, and exploration of genome structure, while the TS/TV ratio of 2.01 indicated a well-balanced and relatively conserved genome structure (25), providing a reliable dataset for future studies on population structure and selection signals.

For this study, we analyzed the population structure and genetic background of three Tibetan sheep populations from different altitudes using PCA, an N-J tree, and ADMIXTURE analysis. Firstly, PCA was used to investigate the clustering of the selected samples. The three populations were relatively close in genetic space, with the branches of the WT and HB populations being close, while the TS population was farther away, which is consistent with the results of the N-J tree. Because PCA and N-J tree are often used in conjunction with model-based clustering methods, we also employed ADMIXTURE analysis on the selected samples. Cross-validation error rates were then compared across different K values, revealing a K value of 2 as the optimal modeling choice. The TS population exhibited separation from the other two, with a predominantly dark blue background. As the K value was increased, the population structure exhibited a mixed state with a diverse genetic composition and relatively complex origins. Our findings point to obvious gene flow among the different Tibetan sheep populations, which is consistent with their geographical locations. The results obtained through mutual verification of the three methods were consistent, indicating the reliability of the data.

Altitude adaptation is a complex process resulting from the combined action of multiple genes and pathways (26). When individuals live at high-altitudes for an extended period, their bodies undergo a series of adaptive changes to maintain normal physiological functions and stabilize the internal environment. These changes involve multiple mechanisms such as energy metabolism, hypoxic response, and cardiovascular function (27). In this study, altitude adaptation-related genes were found to be mainly concentrated in metabolic pathways (*GSTA1*, *ALAS1*, *HMOX2*, *SCD*, *ME1*, *ACSL6*, *PIK3C2G*), melanogenesis (*MITF*, *EP300*), and the HIF-1 signaling pathway (*HIF1A*, *ERBB2*, *RELA*). The hypoxic conditions of high-altitude environments can lead to mitochondrial dysfunction and increased oxidative stress (28, 29). *GSTA1* (Glutathione S-transferase alpha 1) is a widely expressed enzyme that plays a central role in alleviating oxidative stress and detoxification, particularly in the process of scavenging hydrogen peroxide (30, 31). The gene expression and activity of *GSTA1* are modulated under hypoxic conditions, potentially helping cells adapt to low-oxygen environments through its antioxidant and detoxification functions. Heme is a cofactor for various proteins (e.g., hemoglobin) and plays an important role in multiple processes, including oxygen transport, electron transfer, and detoxification. The activity of the rate-limiting enzyme in heme synthesis, *ALAS1* (5-aminolevulinate synthase 1), directly affects heme production, which is crucial for oxygen transport and uptake. *HMOX2* (Heme oxygenase 2) plays an important role in heme metabolism, catalyzing the decomposition of heme into carbon monoxide (CO), a crucial gaseous signaling molecule. CO plays a significant role in regulating vascular relaxation and oxidative stress, thereby influencing oxygen utilization and cellular metabolism (32). Additionally, *HMOX2* also plays a key role in angiogenesis regulation, particularly in maintaining endothelial cell function and vascular stability (33). The enzyme encoded by the *SCD* (Stearoyl-CoA desaturase) gene catalyzes the conversion of saturated fatty acids to monounsaturated fatty acids. In the plateau lizard *Phrynocephalus vlangalii* (34), upregulated expression of *SCD* promotes fatty acid synthesis and storage, helping larvae adapt to high-altitude environments. *ME1* (Malic enzyme 1) is a key metabolic enzyme that catalyzes the conversion of malate to pyruvate while generating NADPH (35), an important cellular reducing agent that participates in various biosynthetic and antioxidant reactions. In high-altitude, low-oxygen environments, the activity of *ME1* is thought to affect the cellular redox state and metabolic pathways by regulating the NADPH/NADP+ ratio (36, 37). *ACSL6* (Acyl-CoA synthetase long chain family member 6), a key enzyme for the uptake and transport of free fatty acids, regulates the expression of a series of downstream genes involved in lipid metabolism (38). *ACSL6* has been associated with lipid metabolism under hypoxic stress (39), suggesting that adaptation to low-oxygen environments may depend on enhanced fatty acid oxidation. Additionally, a study compared copy number variations between high-altitude and low-altitude cattle breeds in Ethiopia. It was found that the *PIK3C2G* (Phosphatidylinositol-4-phosphate 3-kinase catalytic subunit type 2 gamma) gene exhibited significant differentiation in high-altitude cattle breeds (40). This finding in cattle, further supported by similar observations in pigs (41), suggests that *PIK3C2G* may be associated with high-altitude adaptation, and indicates its possible importance in Tibetan sheep adaptation to high-altitude, low-oxygen environments. Collectively, these genes appear to play crucial roles in enabling organisms to effectively cope with the challenges posed by low-oxygen environments through the regulation of various adaptive mechanisms.

Generally, UV radiation is stronger at high-altitudes, potentially leading to DNA damage in skin tissues. In this study, we found that the *MITF* (Microphthalmia-associated transcription factor) gene is significantly enriched in the melanogenesis pathway, which is related to UV resistance (42), with similar research conducted in Tibetan goats (4). Hu et al. (2) found that *MITF* may be a candidate gene for high-altitude adaptation in sheep and plays a significant role in the adaptive gradual infiltration from argali to Tibetan sheep (43). Alternatively, populations residing at high altitudes have been shown to exhibit strong positive selection signals for the *EP300*(E1A binding protein p300) gene, which plays a crucial role in the regulation of sensory cell damage (44). Furthermore, there is a significant correlation between the expression level of *EP300* and the content of nitric oxide (NO) in the blood. Specifically, *EP300* may enhance the adaptability of Tibetan populations to hypoxic environments by increasing the level of NO (44). In summary, *MITF* and *EP300* may contribute to the body’s response to hypoxic challenges in high-altitude environments.

Hypoxia-inducible factor-1 (HIF-1) can be activated in hypoxic environments to influence the expression of many genes, playing a crucial role in regulating the stability of oxygen levels in the body (45). In this study, we identified enrichment of key genes in the HIF-1 signaling pathway that may play pivotal roles in high-altitude adaptation, namely *HIF1A*, *ERBB2*, and *RELA*. HIF1A, the core regulatory factor of the HIF-1 signaling pathway, is transcribed at low levels under normal oxygen conditions, but significantly increases in hypoxic states, which affects erythrocyte production, influencing processes such as cell proliferation and death, and leading to hypoxic adaptive responses (46). The *ERBB2* (Tyrosine kinase receptor-2) gene plays a key regulatory role in the growth and drug resistance of breast cancer. Studies have shown that *ERBB2* relies on HIF-1 to promote breast cancer growth *in vivo*, enhancing the adaptability of tumor cells to hypoxic environments through HIF-1-mediated signaling pathways, thereby generating oxygen resistance (47). Cyclooxygenase-2 (COX-2) is induced by hypoxic environments in vascular endothelial cells, with the induction process mediated by *RELA* (V-rel reticuloendotheliosis viral oncogene homolog A) (48). The interactions among these genes and their synergistic effects in the HIF-1 signaling pathway provide new insights into the molecular mechanisms of high-altitude hypoxic adaptation. However, their specific functions and regulatory networks in high-altitude adaptation require verification through further experimental studies.

## Conclusion

This study reports genome-wide selection signals in Tibetan sheep populations at high, medium, and low altitudes using comprehensive Fst and θπ ratio analyses. Adaptation to high altitudes was found to involve genes associated with metabolic pathways (*GSTA1*, *ALAS1*, *HMOX2*, *SCD*, *ME1*, *ACSL6*, *PIK3C2G*), melanogenesis (*MITF*, *EP300*), and the HIF-1 signaling pathway (*HIF1A*, *ERBB2*, *RELA*). These genes are primarily linked to energy metabolism, angiogenesis, the hypoxic response, and UV protection. Our findings provide data for further exploration of the hypoxic adaptation mechanisms of Tibetan sheep, and serve as a reference for the prevention and treatment of high-altitude illnesses in humans.

## Data Availability

The datasets presented in this study can be found in online repositories. The names of the repository/repositories and accession number(s) can be found at: https://www.ncbi.nlm.nih.gov/, PRJNA1138910.

## Footnotes

^1^ http://sourceforge.net/projects/picard/

^2^ https://itol.embl.de/upload.cgi

^3^ https://david.ncifcrf.gov/

^4^ http://kobas.cbi.pku.edu.cn/kobas3/genelist/

^5^ https://www.ncbi.nlm.nih.gov/
