# Supplementary material for: Genome-wide selection signal analysis reveals the adaptability of Tibetan sheep to high altitudes
Source: Front Vet Sci. 2025 Aug 14;12:1632017. doi: 10.3389/fvets.2025.1632017 (PMC12392279; doi:10.3389/fvets.2025.1632017)
Supplement: Supplementary file 1 [file Supplementary_file_1.docx]

Supplementary Material

## **Supplementary Figures**


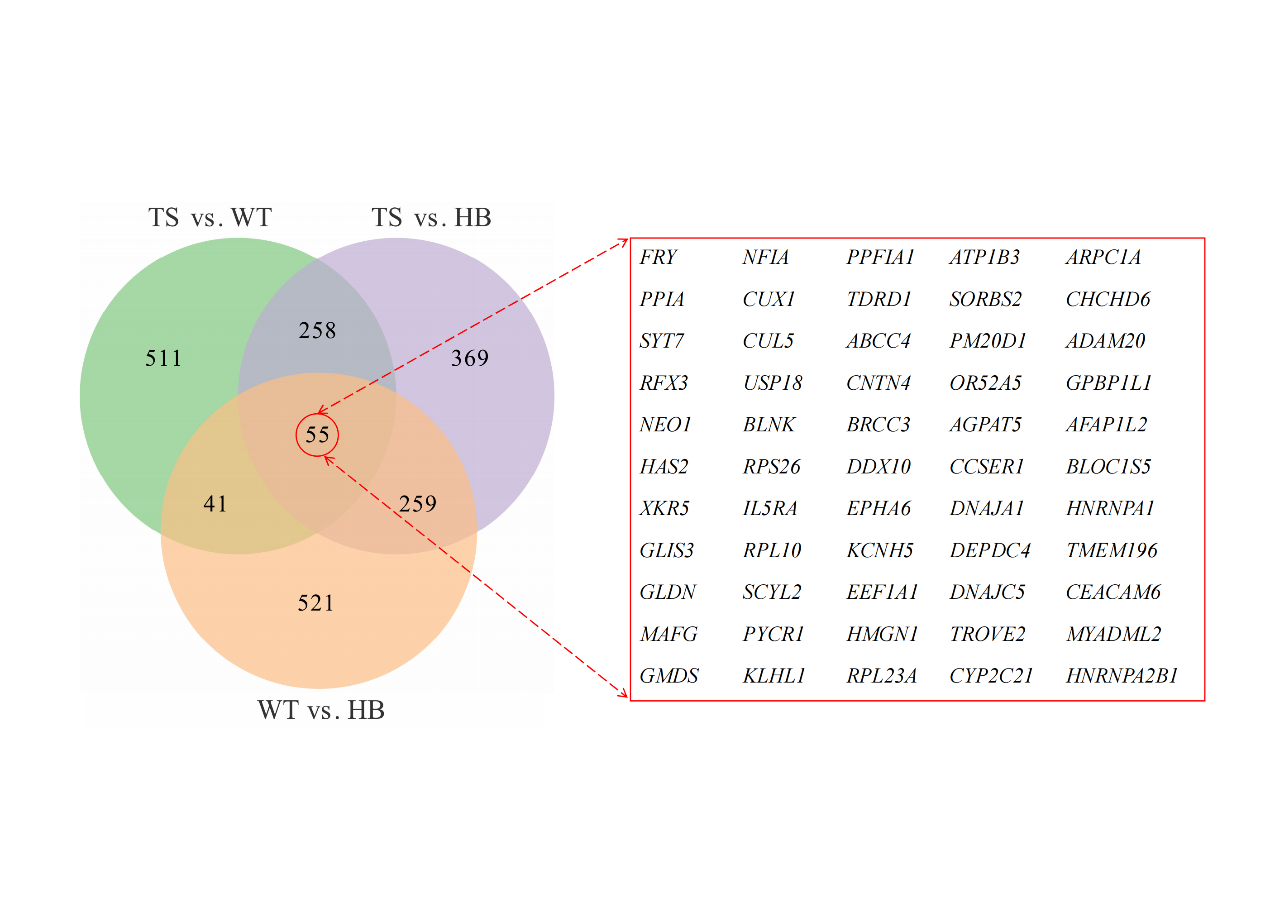


**Supplementary Figure 1.** Venn diagram of candidate genes identified in the three group comparisons (TS vs. WT, TS vs. HB, WT vs. HB).
